# Enhanced Cellular Uptake of Albumin-Based Lyophilisomes when Functionalized with Cell-Penetrating Peptide TAT in HeLa Cells

**DOI:** 10.1371/journal.pone.0110813

**Published:** 2014-11-04

**Authors:** Etienne van Bracht, Luuk R. M. Versteegden, Sarah Stolle, Wouter P. R. Verdurmen, Rob Woestenenk, René Raavé, Theo Hafmans, Egbert Oosterwijk, Roland Brock, Toin H. van Kuppevelt, Willeke F. Daamen

**Affiliations:** 1 Department of Biochemistry, Radboud Institute for Molecular Life Sciences, Radboud university medical centre, Geert Grooteplein 28, 6525 GA, Nijmegen, The Netherlands; 2 Department of Laboratory Medicine, Laboratory of Hematology, Radboud university medical centre, Geert Grooteplein 8, 6525 GA, Nijmegen, The Netherlands; 3 Department of Urology, Radboud Institute for Molecular Life Sciences, Radboud university medical centre, Geert Grooteplein 28, 6525 GA, Nijmegen, The Netherlands; University of Helsinki, Finland

## Abstract

Lyophilisomes are a novel class of biodegradable proteinaceous nano/micrometer capsules with potential use as drug delivery carrier. Cell-penetrating peptides (CPPs) including the TAT peptide have been successfully implemented for intracellular delivery of a broad variety of cargos including various nanoparticulate pharmaceutical carriers. In the present study, lyophilisomes were modified using CPPs in order to achieve enhanced cellular uptake. Lyophilisomes were prepared by a freezing, annealing, and lyophilization method and a cystein-elongated TAT peptide was conjugated to the lyophilisomes using a heterobifunctional linker. Fluorescent-activated cell sorting (FACS) was utilized to acquire a lyophilisome population with a particle diameter smaller than 1000 nm. Cultured HeLa, OVCAR-3, Caco-2 and SKOV-3 cells were exposed to unmodified lyophilisomes and TAT-conjugated lyophilisomes and examined with FACS. HeLa cells were investigated in more detail using a trypan blue quenching assay, confocal microscopy, and transmission electron microscopy. TAT-conjugation strongly increased binding and cellular uptake of lyophilisomes in a time-dependent manner *in vitro*, as assessed by FACS. These results were confirmed by confocal microscopy. Transmission electron microscopy indicated rapid cellular uptake of TAT-conjugated lyophilisomes via phagocytosis and/or macropinocytosis. In conclusion, TAT-peptides conjugated to albumin-based lyophilisomes are able to enhance cellular uptake of lyophilisomes in HeLa cells.

## Introduction

An innovative strategy in cancer therapy utilizes drug delivery carriers to increase the therapeutic effect of anti-tumor drugs. The role of drug delivery carriers in this context is to improve pharmacokinetics and dynamics by protecting the drug from degradation [1]–[3]. Contemporary drug delivery systems include nanoparticulate systems loaded with anti-tumor drug and conjugates directly coupled to the drug. The unique property of nanoparticle carriers is their ability to encapsulate and deliver a high dose of anti-tumor drugs, including poorly soluble drugs, and to exploit the enhanced permeability and retention (EPR) effect for tumor targeting [4]. Because of these favorable characteristics, there has been intense interest in the development of nanoparticulate drug delivery systems.

Nanoparticles currently investigated for cancer therapeutic applications include liposomes, polymersomes, dendrimers, micelles, carbon nanotubes, nanoconjugates and (protein-based) nanospheres or capsules [5]–[7]. Among the available potential drug carrier systems, protein-based nanoparticles are particularly interesting as they hold certain advantages such as good stability during storage, non-toxicity, biocompatibility, and biodegradability *in vivo* [8], [9]. Recently we showed that lyophilisomes, a novel class of proteinaceous biodegradable hollow nano/micrometer capsules, show potential as a drug delivery capsule [10], [11]. Lyophilisomes can be prepared from a large variety of water-soluble macromolecules including proteins (*e.g.* albumin and elastin) but also polysaccharides (*e.g.* heparin). In fact, virtually any biomolecule can be incorporated into the wall/lumen of the capsule, resulting in a highly flexible carrier system with multiple applications. We previously demonstrated that enzymes introduced in the capsule's wall and in the lumen are bioactive and able to convert a substrate [10]. Furthermore, lyophilisomes have been efficiently loaded with doxorubicin resulting in tumor cell elimination *in vitro* [11]. In order to obtain a selective drug delivery system, lyophilisomes have also been modified with antibodies, resulting in specific targeting of the cell of interest *in vitro* [12]. Due to these properties, lyophilisomes can be deployed for the design of multifunctional targeting systems.

Lyophilisomes were prepared from albumin. Albumin is an attractive macromolecular carrier that has been shown to be non-toxic, non-immunogenic, biodegradable to produce innocuous degradation products, and easy to purify [13]. It is thus a suitable candidate for nanoparticle preparation, as demonstrated by FDA-approved products such as Abraxane [14], [15] and Albunex [16], [17].

Since delivery of nanocarriers is generally based on passive accumulation in pathological tissues, they do not efficiently deliver their cargo to specific cells. When drug delivery carriers arrive at the tumor site, they have to cross the plasma membrane in order to deliver the drug into the cell. The plasma membrane, however, prevent proteins, peptides, and nanoparticulate drug carriers from entering the cell in the absence of an active transport mechanism [18]. Cell-penetrating peptides (CPPs) have been successfully used to deliver a large variety of cargos to the cell interior including proteins [19], peptides [20], nucleic acids [21] and pharmaceutical nanocarriers [22]–[24]. CPPs are short peptides consisting of up to 30 amino acids that are able to translocate across the cellular membrane [25]. When CPPs are conjugated to drug delivery carriers, efficient cellular uptake of the carriers can be achieved [26]. A representative CPP is the TAT peptide, derived from the TAT protein (trans-activation transcriptional activator) of the human immunodeficiency virus type 1 (HIV-1) [27], [28]. The TAT peptide consists of 11 amino acids with the sequence YGRKKRRQRRR. The abundance of lysine and arginine residues makes it highly positively charged, important for the interaction with the plasma membrane. In this study, the TAT peptide was conjugated to lyophilisomes to investigate whether CPPs are able to enhance cellular uptake of lyophilisomes.

## Materials and Methods

Bovine serum albumin was purchased from PAA Laboratories (Linz, Austria). FITC-conjugated bovine albumin was purchased from Sigma Aldrich (Steinheim, Germany). Sulfo-GMBS (sulfo-*N*-[γ-maleimidobutyryloxy]sulfosuccinimide ester) was purchased from Pierce Biotechnology (Rockford, IL, USA). Glutaraldehyde and formaldehyde were obtained from Merck (Darmstadt, Germany). Cysteine functionalized TAT peptide (C-Ahx-YGRKKRRQRRR) was purchased from EMC Microcollections GmbH (Tübingen, Germany), in which Ahx  =  aminohexanoic acid linker.

### Preparation of lyophilisomes

Lyophilisomes were prepared from albumin as described previously [11]. Briefly, droplets of a solution of 0.25% (w/v) bovine serum albumin (BSA) in 0.01 M acetic acid were frozen in liquid nitrogen (−196°C). The frozen albumin preparation was incubated at −10 to −20°C for 3 h (annealing step), and subsequently lyophilized. This procedure results in hollow nano/micro spheres (“lyophilisomes”). In order to visualize the lyophilisomes, FITC-conjugated albumin was added to non-labeled albumin (1∶10) in the starting solution. To obtain stabilized lyophilisomes, they were vapor crosslinked with glutaraldehyde and formaldehyde. Generally we prepare 40 ml of a 0.25% BSA solution, which corresponds to 100 mg albumin (2.5 mg/ml). The final lyophilisome population was centrifuged three or four times at low speed (60×*g*; Thermo, Heraeus Fresco 17; Newport Pagnell, Great Britain) to remove large lyophilisomes and sheet-like structures, until no pellet was observed. After this procedure, about 30% of the original weight of lyophilisomes remained. Lyophilisomes (1 mg/ml) were stored in 0.1% (v/v) Tween-20 (Sigma Aldrich, Steinheim, Germany) in phosphate buffered saline (PBS-T, pH 7.4).

### Conjugation of cell-penetrating peptide to lyophilisomes

**Figure 1:**
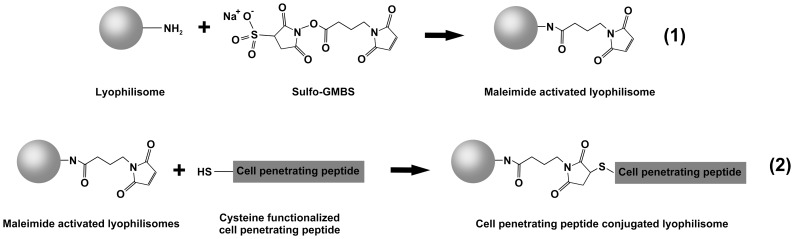
Schematic illustration of the conjugation of the cell-penetrating peptide (CPP) TAT to lyophilisomes. (1) Primary amine groups of lyophilisomes react with Sulfo-GMBS introducing reactive maleimide groups. (2) CPPs (cysteine-functionalized TAT-peptides; C-Ahx-YGRKKRRQRRR) are conjugated to maleimide-conjugated lyophilisomes, resulting in stable CPP-conjugated lyophilisomes. Sulfo-GMBS  =  sulfo-*N*-[γ-maleimidobutyryloxy]sulfo succinimide ester; Ahx  =  aminohexanoic acid; TAT  =  trans-activating transcriptional activator.

A schematic representation of the conjugation reactions is depicted in figure 1.

#### Reaction 1

Activation of lyophilisomes. To obtain maleimide-activated lyophilisomes, 1 mg lyophilisomes were resuspended in 1 ml PBS-T and incubated overnight with 31 µl of 10 mM sulfo-GMBS in PBS-T (pH 8.0) at 4°C on a rotator (36 rpm, “Assistant” Rotating mixer, Karl Hecht, Sondheim, Germany), resulting in a 20∶1 molar ratio of sulfo-GMBS∶albumin. Excess sulfo-GMBS was removed by centrifugation (5 min, 17,000×*g*, 4°C) with three washing steps in PBS-T (pH 6.5).

#### Reaction 2

Conjugation of TAT peptide to lyophilisomes. For the coupling reaction, 1 ml of 1 mg/ml sulfhydryl-reactive lyophilisomes in 0.1% PBS-T was centrifuged and conjugated in 1 ml of cysteine functionalized TAT peptide (100 µM; C-Ahx-YGRKKRRQRRR) in 0.1% PBS-T (pH 6.5). Non-coupled TAT peptides were removed by centrifugation, using three washing steps in 0.1% PBS-T (pH 7.4). TAT-conjugated lyophilisomes were stored at 4°C in the dark.

### Sorting of lyophilisomes by fluorescence-activated cell sorting

Fluorescence-activated cell sorting (FACS) was applied to select for small lyophilisomes (<1,000 nm) using a Coulter Epics Elite flow cytometer (BeckmanCoulter, Miami, FL, USA). Only small FITC-positive lyophilisomes were sorted (for settings, see section “Lyophilisomes sorted by fluorescence-activated cell sorting”). To achieve high sensitivity, gain was set at 20.

### Particle size measurements by qNano

The qNano (Izon, Science Ltd., Burnside, New Zealand) was used to measure particle size distribution of lyophilisomes [11], [29]. To ensure a continuous flow of particles, a pore size of 600–2000 nm was used. Data were analyzed with Izon Control Suite 2.1 software.

### Cell culture

HeLa (ACC 57, DSMZ, Braunschweig, Germany) and OVCAR-3 (#HTB-161, ATCC, LGC Standards GmbH, Wesel, Germany) cells were cultured in RPMI 1640 GlutaMAX medium Gibco (Karlsruhe, Germany) supplemented with 10% (v/v) fetal calf serum (FCS; PAA Laboratories, Pasching, Austria). Caco-2 cells (#HTB-37, ATCC) and SKOV-3 cells (#HTB-77, ATCC) were cultured in DMEM 1640 GlutaMAX medium Gibco supplemented with 20% (v/v) and 10% (v/v/) fetal calf serum, respectively. Cells were cultured in a humidified atmosphere with 5% CO_2_ at 37°C. Subconfluent cells were dissociated with 0.05% trypsin (w/v) in 0.02% ethylenediaminetetraacetic acid (EDTA) (w/v) in PBS (PAA Laboratories) and were maintained as proliferating cultures.

For FACS, cells were stained with the plasma membrane dye PKH26 (Sigma Aldrich, Missouri, USA) [30], [31]. One million cells were incubated in 2 µM PKH26 dye in 500 µl buffer (according to manufacturer's protocol) for 5 min. To stop the staining reaction, 500 µl FCS was added and incubated for 5 min. Subsequently, cells were washed three times with culture medium.

### Cellular binding and internalization of lyophilisomes with and without TAT peptide

To determine whether the TAT peptide can promote the binding and internalization of lyophilisomes with HeLa, OVCAR-3, Caco-2 and SKOV-3 cells were seeded in a 24-well plate (30,000 cells/well in 1 ml medium). Cells were left to adhere overnight and subsequently incubated for 1 h with unsorted lyophilisomes with and without TAT peptide conjugated to them (25 µg/ml). After incubation, cells were washed three times with PBS to remove unbound lyophilisomes and harvested with enzyme-free EDTA solution (PAA Laboratories). Finally, cells were resuspended in 0.2% BSA in PBS and analyzed by FACS (FACSCalibur Becton Dickinson, Breda, Netherlands). Using the appropriate positive and negative controls FACS settings were adjusted. When lyophilisomes did not bind to cells, lyophilisomes were depicted at a fluorescent signal of 10^1^ or below and cells were regarded negative. When lyophilisomes did bind to cells, they showed a fluorescent signal higher than 10^1^. Data were analyzed by FlowJo software (Version 9.4, Treestar, Ashland, OR, USA).

### Internalization studies of lyophilisomes by cells

#### FACS

In order to discriminate between attached and internalized lyophilisomes, a FITC quenching trypan blue assay was used [32]–[34]. Quenching of FITC signal occurs because trypan blue absorbs the light emitted by FITC-labeled lyophilisomes after excitation. The FITC signal of *internalized* lyophilisomes however, is not quenched since trypan blue cannot pass the plasma membrane. The fluorescence remaining after trypan blue quenching must therefore result from internalized lyophilisomes, as only extracellular fluorescence of FITC-lyophilisomes is quenched.

To investigate the cellular uptake of lyophilisomes, PKH26 stained HeLa cells were seeded in a 24-well plate (30,000 cells/well) and left to adhere overnight. Cells were incubated with 500,000 FACS-sorted lyophilisomes with and without TAT peptide for 1 and 4 h. After incubation, cells were washed three times with 0.2% (w/v) BSA in PBS, dissociated with enzyme-free EDTA dissociation buffer, resuspended in 1 ml culture medium and transferred to an eppendorf tube. Subsequently, cells were washed three times with 0.2% BSA-PBS by centrifugation (3 min, room temperature, 100×g) and incubated with 0.5% (w/v) trypan blue for 10 min and washed three times with 0.2% BSA-PBS. Cells were analyzed by FACSCalibur flowcytometry. Data were analyzed by FlowJo Software.

#### Confocal microscopy

To visualize cellular uptake of lyophilisomes, confocal microscopy was performed on living cells. HeLa cells were seeded in an 8-well microscopy chamber (Nunc; 30,000 cells/well) and left to adhere overnight. Cells were incubated with sorted lyophilisomes with and without TAT peptide using 0.8 and 3.5 million lyophilisomes in 200 µl per sample for 4 h in RPMI medium containing 10% FCS at 37°C. As a control, medium without lyophilisomes was used. After incubation, cells were washed three times, incubated for 5 min with CellMask Orange (5 µg/ml) to visualize the plasma membranes and then washed again, all with the same medium. Cells were kept at 37°C on a temperature controlled microscope stage and living cells were imaged immediately with a Leica SP5 confocal microscope (Leica Microsystems, Mannheim, Germany). FITC was excited at 488 nm and emission was collected between 500–550 nm. CellMask orange was excited at 561 nm and emission was collected between 570–650 nm. Images were recorded sequentially using Leica Application Suite Software (Advanced Fluorescence Lite, 2.3.0. build 5131).

#### Transmission electron microscopy

Cells incubated with lyophilisomes with and without TAT peptide as described in the Materials and Methods section “*FACS*” were embedded in 1.5% (w/v) agarose, fixed in 2% (v/v) glutaraldehyde in 0.1 M phosphate buffer (pH 7.4), post-fixed with 1% (w/v) osmium tetroxide, dehydrated in an ascending series of ethanol, and embedded in Epon 812. Ultrathin sections (60 nm) were cut and picked up on Formvar-coated grids, post-stained with lead citrate and uranyl acetate, and examined with a JEOL 1010 transmission electron microscope (Tokyo, Japan).

### Statistical Analysis

Data are presented as mean with standard deviation. Data of Results section “*Cellular binding and internalization of lyophilisomes with and without TAT peptide*” were analyzed using two-tailed Student's *t*-tests. Data of Results section “*Trypan blue assay and FACS*” were analyzed using two-way Anova Bonferroni post-hoc tests. All statistical analyses were performed in Graphpad Prism 5.0 (Graphpad, San Diego, CA, USA). P values<0.05 were considered significant.

## Results

### Conjugation of TAT peptide to lyophilisomes

To probe the possibility of using CPP for enhanced intracellular delivery, lyophilisomes were modified with a cysteine-functionalized TAT peptide using the heterobifunctional linker sulfo-GMBS (Fig. 1). The succinimidyl ester functionality is conjugated to primary amine groups on the lyophilisome while the maleimide functionality is used for conjugation to the free thiol of the cysteine residue coupled to the TAT peptide.

### Cellular binding and internalization of lyophilisomes with and without TAT peptide

**Figure 2:**
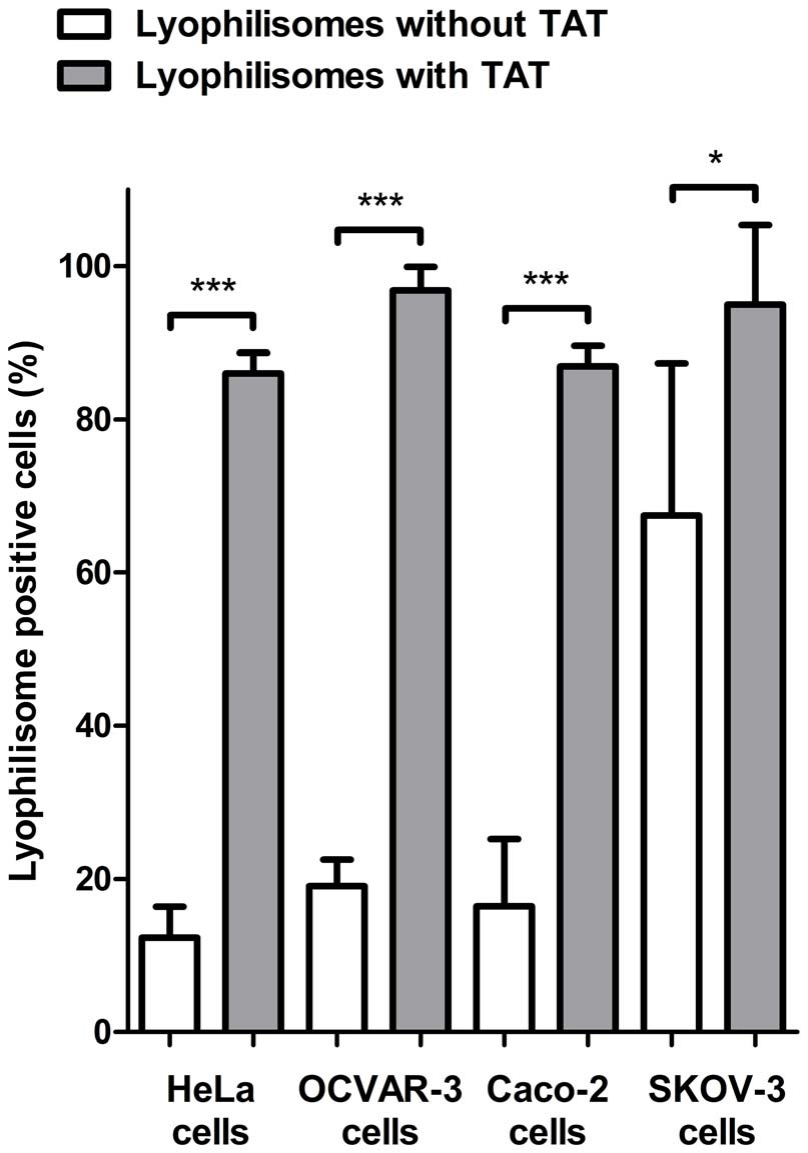
Cellular binding and internalization of unmodified lyophilisomes and TAT-conjugated lyophilisomes. HeLa, OVCAR-3, Caco-2 and SKOV-3 cells incubated with TAT-conjugated and unmodified lyophilisomes resulted in 86±3% and 12±4%, 87±3% and 16±8%, 97±3% and 19±3%, and 95±10% and 67±20% lyophilisome-positive cells, respectively. TAT  =  trans-activating transcriptional activator. *p<0.01 ***p<0.0001.

To address the presence of the TAT peptide on TAT-conjugated lyophilisomes, unmodified lyophilisomes and TAT-conjugated lyophilisomes were administered to HeLa, OVCAR-3, Caco-2 and OVCAR-3 cells. Using standard FACS as a functional assay, it is not possible to discriminate between cellular attachment and internalization. Instead, the total of cell binding and internalization is measured (Fig. 2). For HeLa cells, TAT-conjugated lyophilisomes showed an about 8-fold increase in lyophilisome-positive cells compared to lyophilisomes without the TAT peptide (86±3% and 12±4% lyophilisome-positive cells for TAT-conjugated and unmodified lyophilisomes, respectively. OVCAR-3 and Caco-2 cells showed about a 5-fold increase in lyophilisome-positive cells compared to lyophilisomes without the TAT peptide (lyophilisome-positive cells: 97±3% and 19±3% for OVCAR-3; 87±3% and 16±8% for Caco-2) for TAT-conjugated and unmodified lyophilisomes, respectively. SKOV-3 cells gave a high background value when incubated with lyophilisomes without TAT (67±20%), but still showed a 1.6 fold statistically significant increase with the presence of TAT peptide (95±10%).

### Lyophilisomes sorted by fluorescence-activated cell sorting

**Figure 3:**
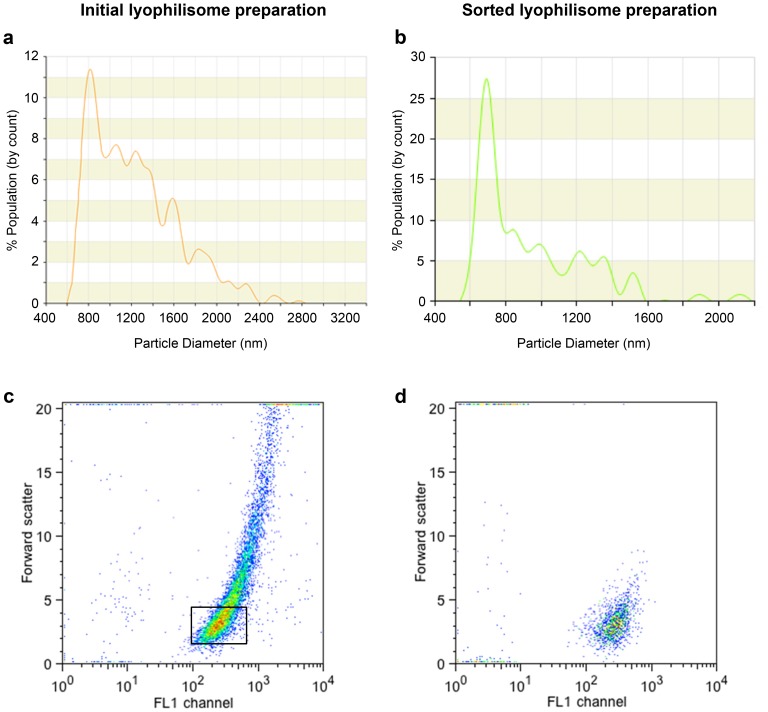
Sorting of lyophilisomes by fluorescence-activated cell sorting. a/b) A representative size distribution of the initial lyophilisome population (a) and sorted lyophilisomes (b) is depicted, showing smaller lyophilisomes after sorting. Note the difference in x and y axes. c) Initial lyophilisome population depicted in a FACS dot plot with forward (size)/FITC-positive lyophilisome (FL1 channel) scatter where gated FITC-positive lyophilisomes were sorted. d) After sorting, the scatter showed merely small lyophilisomes, as large lyophilisomes were removed.

When using lyophilisomes for tumor targeting, lyophilisomal size is an important parameter. The initial lyophilisome population included sizes up to 2.8 µm (Fig. 3a). To obtain a more monodisperse capsule population, lyophilisomes were sorted by FACS. Lyophilisomes were separated based on forward scatter and FITC fluorescence (FL1 channel; Fig. 3c). To verify the procedure, a rerun of sorted lyophilisomes was performed (Fig. 3d). Using this methodology, larger lyophilisomes as well as sheet-like structures (confirmed by scanning electron microscope) were separated from small lyophilisomes. These results were substantiated by qNano size analysis using a lyophilisome preparation before (Fig. 3a) and after (Fig. 3b) sorting (approximately 90% below 1 µm). Measurements using the qNano consisted of 200–250 particles. The size distribution contained multiple peaks that can be explained by the low number of particles. Due to the limitations of the qNano instrument particles smaller than 600 nm could not be reliably detected which overestimates the average size of the lyophilisomes. Pilot experiments with a smaller qNano pore (200–800 nm) revealed the presence of lyophilisomes below 600 nm (results not shown), but larger lyophilisomes in the preparation frequently blocked this pore.

### Cellular uptake of TAT-conjugated lyophilisomes

#### Trypan blue assay and FACS

**Figure 4:**
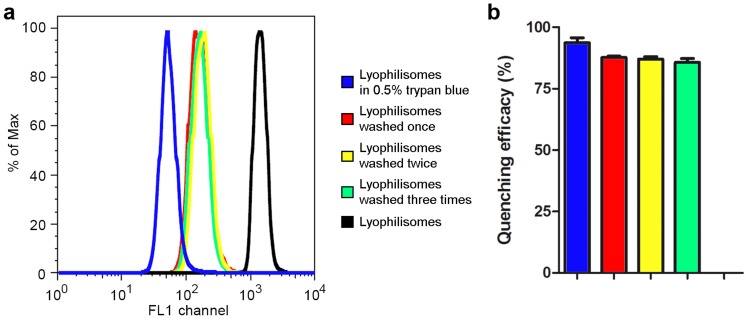
Quenching of FITC fluorescent lyophilisomes by trypan blue in the absence of cells. Results show decreased fluorescence (a) and efficient quenching (b) up to three washings steps of FITC fluorescent lyophilisomes by trypan blue.

To determine the internalization efficiency of sorted lyophilisomes with and without TAT peptide, a trypan blue quenching assay was used in order to distinguish between internalized and non-internalized (but plasma membrane-associated) lyophilisomes. This assay is based on the quenching of fluorescence of FITC-labeled lyophilisomes by the vital stain trypan blue (which does not penetrate plasma membranes). To validate that trypan blue also quenches fluorescence of FITC labeled lyophilisomes, lyophilisomes were incubated with trypan blue in the absence of cells and evaluated by FACS (Fig. 4). Lyophilisomes that were not incubated with trypan blue showed a mean fluorescence intensity of 1339±252. Lyophilisomes incubated in a 0.5% trypan blue solution gave a mean fluorescent intensity of 85±31, corresponding to a quenching efficacy of 94±2% (Fig. 4b). After one, two and three washings after the trypan blue incubation, the measured mean fluorescence was 161±25, 176±22, and 192±19 or 88±1%, 87±1% and 85±1% quenching efficacy, respectively. This indicates that trypan blue was not easily washed out of the lyophilisomes and fluorescence remained quenched. This is important as three washings steps were used prior to FACS analysis.

**Figure 5:**
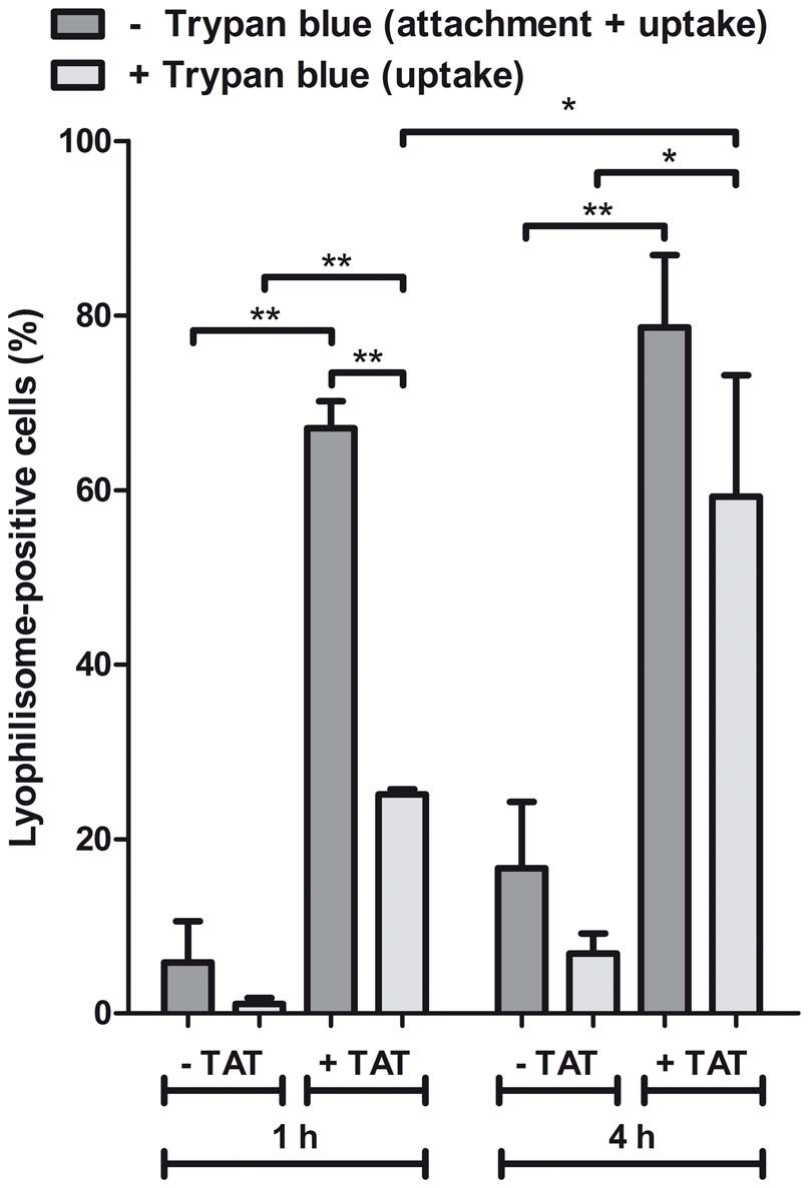
Internalization of lyophilisomes with and without TAT peptide into HeLa cells. FACS showed a large number of lyophilisome-positive cells for TAT-conjugated lyophilisomes after 1 h (67±3%) without trypan blue and a cellular uptake of 25±1% with trypan blue. Values for lyophilisomes without TAT peptide were low. When lyophilisomes were incubated for 4 h, TAT-conjugated lyophilisomes conserved the large number of lyophilisome-positive cells (79±8%) with an increased internalization of 59±14%, while unmodified lyophilisomes still showed few lyophilisome-positive cells and little cellular uptake. *p<0.01 **p<0.001. CPP  =  cell penetrating peptide; TAT  =  trans-activating transcriptional activator.

To investigate whether TAT peptides can enhance internalization of sorted lyophilisomes, the trypan blue quenching assay was performed in the presence of HeLa cells (Fig. 5). Cells were incubated with 500,000 sorted lyophilisomes with and without TAT peptide. Incubation for 1 h with TAT-conjugated lyophilisomes resulted in many lyophilisome-positive cells (67±3%) and moderate cellular uptake (25%±1). In contrast, unmodified lyophilisomes showed few lyophilisome-positive cells (6±5%) and almost no internalization (1±1%). Interestingly, when lyophilisomes were incubated for 4 h, the number of lyophilisome-positive cells of TAT-conjugated lyophilisomes remained high (79±8%) and cellular uptake strongly increased (59±14%), whereas unmodified lyophilisomes still showed a moderate number of lyophilisome-positive cells (17±8%) and little internalization (7±2%).

#### Confocal microscopy

**Figure 6:**
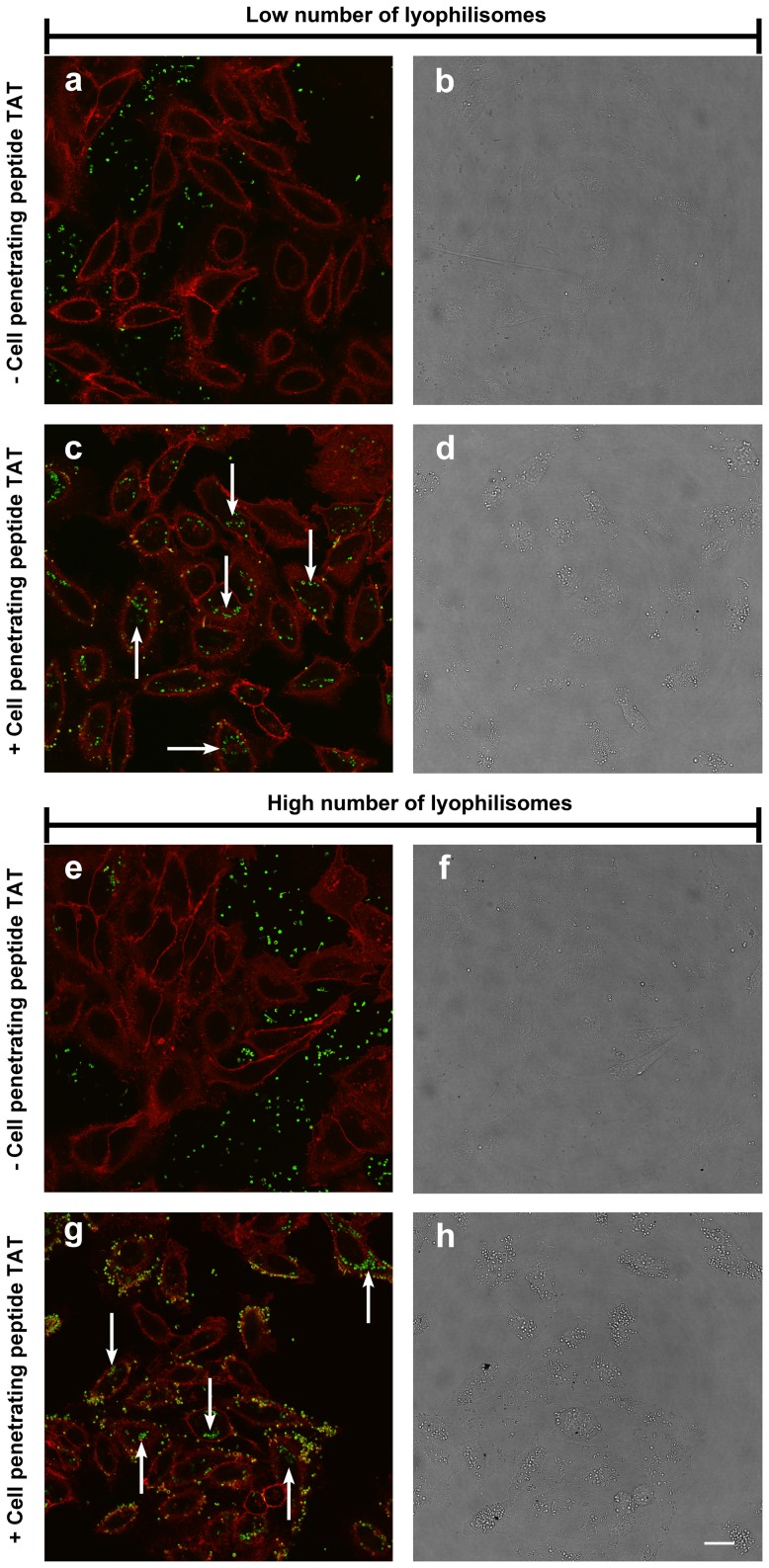
Internalization of lyophilisomes without and with TAT peptide by HeLa cells. Lyophilisomes without and with TAT peptide were administered to 30,000 HeLa cells using two dosages (0.8 million/500 µl (a-d) and 3.5 million/500 µl (e-h). Cells incubated with lyophilisomes without TAT peptide had internalized few lyophilisomes (a/e), whereas TAT-conjugated lyophilisomes showed high cellular uptake for almost all cells (c/g). Green fluorescence corresponds to lyophilisomes and red fluorescence (CellMask orange) visualizes the plasma membrane. Bright field images (b/d/f/h) show normal morphology of the cells. Scale bar represents 20 µm. TAT  =  trans-activating transcriptional activator.

Internalization of TAT-conjugated lyophilisomes in HeLa cells was visualized by confocal microscopy (Fig. 6). After 4 h of incubation, TAT-conjugated lyophilisomes showed extensive uptake for both low (0.8 million/200 µl) and high (3.5 million/200 µl) numbers of lyophilisomes (Fig. 6c/g). Almost no internalization was observed when HeLa cells were incubated with unmodified lyophilisomes (Fig. 6a/e). The corresponding bright field images showed that lyophilisomes did not lead to detectable morphological changes of the cells (Fig. 6b/d/f/h).

#### Transmission electron microscopy

**Figure 7:**
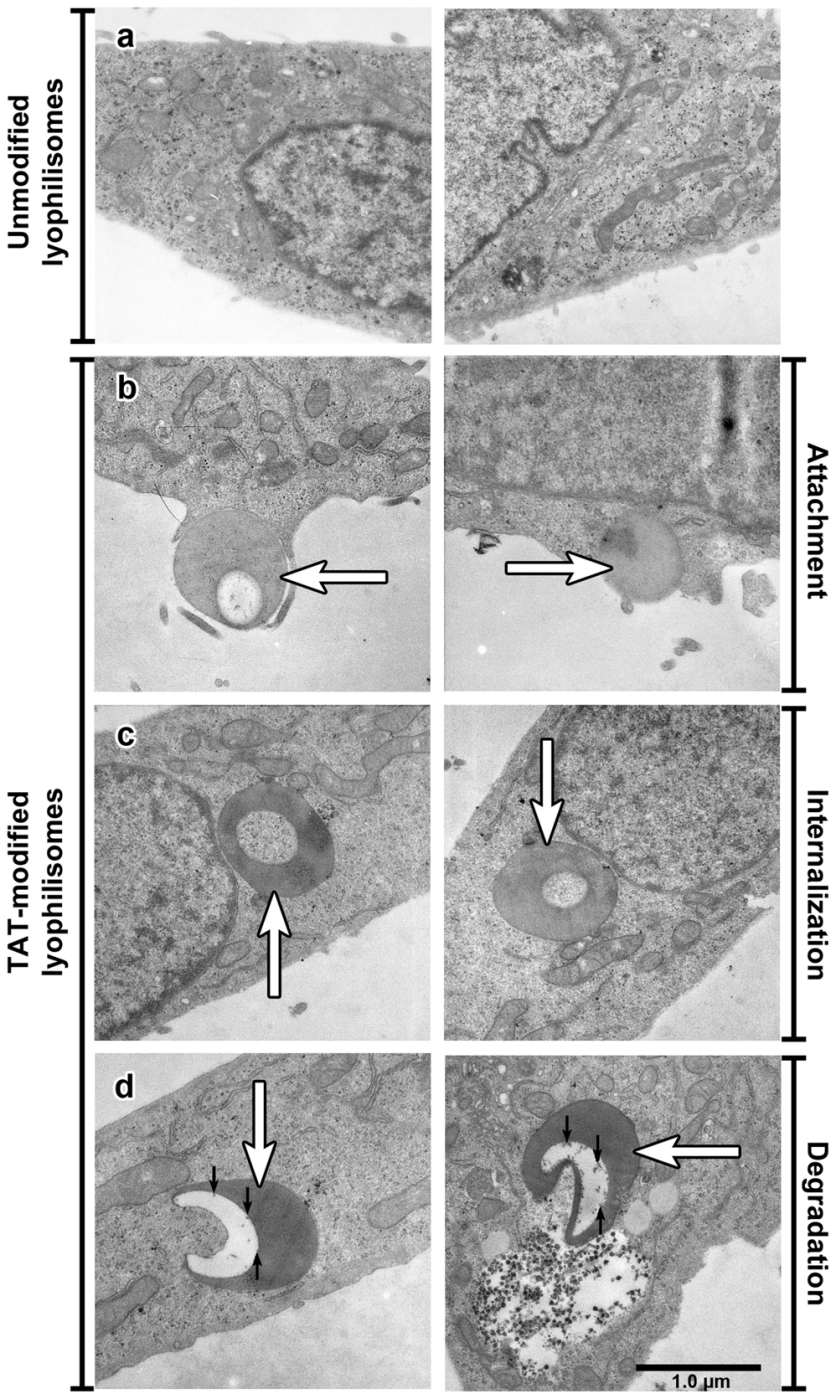
Cellular uptake of TAT-conjugated lyophilisomes as analyzed by transmission electron microscopy. HeLa cells were incubated with unmodified (a) and TAT-conjugated lyophilisomes (b-d) for 4 h. a) No attachment or uptake was observed using unmodified lyophilisomes. b-d) TAT-conjugated lyophilisomes (white arrows) showed various processes required for effective drug delivery systems, such as attachment (b) and uptake (c). Additionally, signs of degradation of the capsule inside the cell were visualized (black arrows, d). Scale bar represents 1.0 µm. TAT  =  trans-activating transcriptional activator.

In order to investigate the binding and uptake of TAT-conjugated lyophilisomes in HeLa cells in detail, TEM was used (Fig. 7). When unmodified lyophilisomes were added to HeLa cells, no binding or uptake was observed and the plasma membrane appeared largely unruffled (Fig. 7a). However, when TAT-conjugated lyophilisomes were added, multiple stages of internalization could be distinguished, including attachment and internalization (Fig. 7b/c). Initially, TAT-conjugated lyophilisomes bound to HeLa cells and initiated membrane ruffling (Fig. 7b). Subsequently, capsules were internalized (Fig. 7c). Furthermore, initial signs of degradation of lyophilisomes could be observed, as degradation products were visible (black arrows; Fig. 7d).

## Discussion

Our laboratory previously demonstrated that lyophilisomes show potential as a drug delivery system [11], [12]. To further optimize these biocapsules, we studied the effect of TAT-functionalization on the *in vitro* internalization of lyophilisomes. Intracellular delivery of therapeutic molecules is one of the key problems in drug delivery. Many pharmaceutical compounds have to be delivered intracellularly to exert their therapeutic action [22]. CPPs have been shown to act as a powerful transport vector for inducing the cellular uptake of a large variety of cargos [18]. At present, pharmaceutical nanocarriers are much in focus for their capacity to increase the stability of administered drugs, improve their concentration at their site-of-action and decrease undesired side effects. Various studies report increased uptake and specific delivery to intracellular organelles when conjugating CPPs to drug delivery systems, thereby increasing the efficiency of nanocarriers as drug delivery systems [35]–[37].

As previously reported, lyophilisomes range in size from 100 up to 3,000 nm in diameter [11]. In this study, we demonstrated that using FACS, small lyophilisomes could be sorted out of the initial population, narrowing the size distribution.

In the present study, FACS was used to investigate the total cell binding and internalization of (TAT-conjugated) lyophilisomes in HeLa, OVCAR-3, Caco-2, and SKOV-3 cells. To investigate the cellular uptake and subcellular distribution of (TAT-conjugated) lyophilisomes in HeLa cells in more detail, confocal microscopy, and TEM were utilized. FACS demonstrated a high number of lyophilisome-positive cells when incubated with TAT-conjugated lyophilisomes compared to unmodified lyophilisomes. This may be explained by interaction of the TAT peptide with negatively charged sulfated glycans at the cell surface, such as heparan sulphate [38], [39]. Only SKOV-3 cells showed a high background when incubated with unmodified lyophilisomes, which has been shown for other drug carriers added to this cell line [40].

To discriminate between internalization and attachment, a trypan blue quenching assay was applied on HeLa cells incubated with lyophilisomes. This assay showed that cellular uptake was enhanced when TAT peptides were conjugated to lyophilisomes. The degree of internalization may even have been underestimated as our capsules are likely to enter the acidic lysosomes in the cell [41] and since FITC fluorescein is virtually non-fluorescent below pH 5 [42]. The trypan blue assay also demonstrated that cellular uptake of TAT-conjugated lyophilisomes increased over time. After 1 h, 25±1% of the cells internalized TAT-conjugated lyophilisomes, whereas 59±14% of cells had done so after an incubation period of 4 h. We most likely added too little lyophilisomes in the trypan blue internalization study to achieve lyophilisome internalization of all available cells. This is supported by confocal microscopy, which revealed that almost all cells internalized at least one lyophilisome when administering more lyophilisomes. TEM images showed different stages of cellular uptake (attachment and internalization) of TAT-conjugated lyophilisomes. The results strongly suggest that TAT-conjugated lyophilisomes are internalized by phagocytosis and/or macropinocytosis, as intensive plasma membrane ruffling was observed during uptake. This suggestion would be in line with the particle size of lyophilisomes [43]. However, lyophilisomes smaller than 200 nm, could still be internalized by one of the mechanisms of endocytosis.

If we compare our findings to other studies, the increased internalization efficiency compared to unmodified particles is within the range of reported values for TAT peptides conjugated to cargos such as liposomes [44], [45]. However, it is difficult to compare internalization for different kinds of nanoparticles, since different cell types and different amounts and sizes of nanoparticles are used for *in vitro* experiments, *e.g.* the larger the particle the more time it takes to establish internalization [46].

The ability of CPPs to enhance cellular uptake non-specifically and receptor-independently provides the opportunity to target diverse cell types with a variety of carriers. In literature, it has been observed that next to HeLa, OVCAR-3, Caco-2 and SKOV-3 cells, the TAT peptide can enter other tumor cells, for instance bladder cancer (HTB-9, MBT2), breast cancer (SK-BR-3, MCF7), and other colon cancer (C26) cells [45], [47]. However, their non-specificity presents a significant challenge in systemically administered applications for targeted delivery, as it requires precise control of CPP presentation only at the target site to prevent toxicity [48]. To overcome this problem, several approaches are being investigated to activate CPPs only at the target site. Stimulus-responsive materials may be used for this purpose, as they may provide triggered changes in material properties that allow spatially focused presentation of CPPs in response to intrinsic disease characteristics (*e.g.* abundantly present extracellular matrix proteases) or locally applied extrinsic cues (*e.g.* apply heat or light at a specific location) [49].

As demonstrated in this study, TAT peptides can enhance cellular uptake when conjugated to lyophilisomes. In previous *in vitro* studies, lyophilisomes were loaded with anti-tumor drugs, *e.g.* doxorubicin and curcumin, which could eliminate tumor cells. Antibodies were conjugated to lyophilisomes resulting in specific binding to target cells [11], [12]. To probe the possibility of using lyophilisomes for the treatment of cancer, active targeting with specific antibodies and enhanced cellular uptake with CPPs can be combined with the drug delivery properties of lyophilisomes, thereby creating a potential powerful tool for drug delivery.

## Conclusion

In the present study, albumin-based lyophilisomes were functionalized with TAT peptides to obtain a drug delivery system with enhanced cellular uptake. Lyophilisomes modified with TAT peptides efficiently bound to HeLa, OVCAR-3, Caco-2 and SKOV-3 cells. Additional cellular uptake studies were performed to verify that TAT-conjugated lyophilisomes were internalized in HeLa cells after binding. TAT-conjugated lyophilisomes may present a novel delivery system to ensure faster and higher cellular uptake of anti-tumor drugs to cancer cells.
