# Correction: Enhanced Cellular Uptake of Albumin-Based Lyophilisomes when Functionalized with Cell-Penetrating Peptide TAT in HeLa Cells

**DOI:** 10.1371/journal.pone.0124465

**Published:** 2015-04-09

**Authors:** The PLOS ONE Staff

The first author’s name is spelled incorrectly in the citation. The correct citation is: van Bracht E, Versteegden LRM, Stolle S, Verdurmen WPR, Woestenenk R, Raavé R, et al. (2014) Enhanced Cellular Uptake of Albumin-Based Lyophilisomes when Functionalized with Cell-Penetrating Peptide TAT in HeLa Cells. PLoS ONE 9(11): e110813. doi:10.1371/journal.pone.0110813
